# Telehealth Care for Mothers and Infants to Improve the Continuum of Care: Protocol for a Quasi-Experimental Study

**DOI:** 10.2196/41586

**Published:** 2022-12-15

**Authors:** Kimiyo Kikuchi, Rafiqul Islam, Yoko Sato, Mariko Nishikitani, Rieko Izukura, Nusrat Jahan, Fumihiko Yokota, Subaru Ikeda, Nazneen Sultana, Meherun Nessa, Morshed Nasir, Ashir Ahmed, Kiyoko Kato, Seiichi Morokuma, Naoki Nakashima

**Affiliations:** 1 Department of Health Sciences Graduate School of Medical Sciences Kyushu University Fukuoka Japan; 2 Medical Information Center Kyushu University Hospital Fukuoka Japan; 3 Social Medicine, Department of Basic Medicine Faculty of Medical Sciences Kyushu University Fukuoka Japan; 4 Grameen Communications Dhaka Bangladesh; 5 Institute for Asian and Oceanian Studies Kyushu University Fukuoka Japan; 6 Holy Family Red Crescent Medical College & Hospital Dhaka Bangladesh; 7 Faculty of Information Science and Electrical Engineering Kyushu University Fukuoka Japan; 8 Department of Obstetrics and Gynecology Graduate School of Medical Sciences Kyushu University Fukuoka Japan

**Keywords:** telehealth care, continuum of care, maternal, newborn, and child health, portable health clinic, parenting, prenatal, pediatrics

## Abstract

**Background:**

Ensuring an appropriate continuum of care in maternal, newborn, and child health, as well as providing nutrition care, is challenging in remote areas. To make care accessible for mothers and infants, we developed a telehealth care system called *Portable Health Clinic for Maternal, Newborn, and Child Health*.

**Objective:**

Our study will examine the telehealth care system’s effectiveness in improving women’s and infants’ care uptake and detecting their health problems.

**Methods:**

A quasi-experimental study will be conducted in rural Bangladesh. Villages will be allocated to the intervention and control areas. Pregnant women (≥16 gestational weeks) will participate together with their infants and will be followed up 1 year after delivery or birth. The intervention will include regular health checkups via the Portable Health Clinic telehealth care system, which is equipped with a series of sensors and an information system that can triage participants’ health levels based on the results of their checkups. Women and infants will receive care 4 times during the antenatal period, thrice during the postnatal period, and twice during the motherhood and childhood periods. The outcomes will be participants’ health checkup coverage, gestational and neonatal complication rates, complementary feeding rates, and health-seeking behaviors. We will use a multilevel logistic regression and a generalized estimating equation to evaluate the intervention’s effectiveness.

**Results:**

Recruitment began in June 2020. As of June 2022, we have consented 295 mothers in the study. Data collection is expected to conclude in June 2024.

**Conclusions:**

Our new trial will show the effectiveness and extent of using a telehealth care system to ensure an appropriate continuum of care in maternal, newborn, and child health (from the antenatal period to the motherhood and childhood periods) and improve women’s and infants’ health status.

**Trial Registration:**

ISRCTN Registry ISRCTN44966621; https://www.isrctn.com/ISRCTN44966621

**International Registered Report Identifier (IRRID):**

DERR1-10.2196/41586

## Introduction

In Sustainable Development Goal 3, maternal, newborn, and child health (MNCH) were identified as essential issues for the world to address [1]. A vital and global framework in improving mothers’, newborns’, and children’s health status is ensuring the continuum of care in MNCH within resource-limited settings. The continuum of care is the series of care throughout the adolescence and prepregnancy periods, the antenatal period, and the motherhood and childhood periods. Further, in terms of the nature of care, the continuum of care spans across community care, primary care, and advanced care [2-4]. Comprehensively monitoring infants’ nutrition status has also been emphasized in the context of the continuum of care in MNCH [5]. To ensure infants’ physical and psychological development and long-term health, they must receive appropriate care at the right times.

In this context, telehealth care is gaining attention, as it can be used to fill the gaps in the continuum of care [6]. Telehealth care may have the potential to monitor and provide care for high-risk pregnancies [7,8]. According to previous studies, telehealth care provided through either the internet or the telephone for the treatment of postpartum depression showed favorable effects thereon (mean difference: −1.81, 95% CI −2.68 to −0.93) [9]. Further, by using telehealth care monitoring, women with gestational diabetes mellitus showed an improvement in hemoglobin A1c levels (−0.41%, 95% CI −0.25% to −0.04%) when compared with a control group [10]. Another study on gestational diabetes mellitus found lower incidence rates of cesarean sections and maternal and neonatal complications in the telehealth care group [11]. Thus, the need for telehealth care follow-ups is sure to gain importance in the future.

In Bangladesh, the MNCH status has been improving. However, the maternal and neonatal mortality rates in Bangladesh are still among the highest in Asia; 173 maternal deaths per 100,000 live births and 19.1 neonatal deaths per 1000 live births were reported in 2019 [12]. As a considerable number of maternal and neonatal deaths and gestational complications are avoidable through early health intervention, providing appropriate and continuous care is essential. However, only 44% of women receive antenatal care (≥4 times) from a medically trained health care provider, and 47% of women and 46% of newborns do not receive any postnatal care [13], indicating inadequate MNCH services in Bangladesh. Further, Bangladesh’s cesarean section rate (30.7%) [12], which is higher than the global mean, may be a consequence of insufficient health care, as the cesarean section rate is often considered a proxy indicator of women’s access to care. Several factors have been identified that relate to the lower utilization of MNCH care services, including family wealth, previous experiences of childbirth, autonomy in women [14], and the distance to health facilities [15]. Therefore, financially and physically affordable care needs to be explored further to improve access to care.

Infants’ development status has also been recognized as a problem in Bangladesh; 28% of newborns had a low birth weight in 2015 [16], which was the highest low birth weight rate among newborns in the world at the time. Accordingly, undernutrition in 5- to 9-year-old children was also reported, with 18% of children categorized as *thin* (BMI of <−2 SDs below the mean)—the third highest undernutrition rate among 5- to 9-year-old children in the world. Anemia was also detected among 43% of children aged 6 to 59 months [12]. Hence, children’s nutrition status is also an essential aspect that needs to be monitored at and after birth.

The Portable Health Clinic (PHC) is a telehealth care system that comprises a set of sensor devices in an attaché case and an information system that can automatically triage health levels once users input the results of a checkup [17]. Originally developed in collaboration with Grameen Communications and Kyushu University, the PHC has been used in Bangladesh for telehealth checkups with over 45,000 people to prevent lifestyle diseases, such as diabetes and hypertension. Receiving care at home is beneficial for people who live in remote areas where access to health care is limited. Later, the MNCH module—PHC for Maternal, Newborn, and Child Health (PHC-MNCH)—was developed and piloted in rural areas of Bangladesh to provide antenatal and postnatal health checkups [18,19]. The PHC-MNCH has been renewed, and its original coverage (16 gestational weeks to 6 weeks after birth) has been expanded to include follow-ups at 1 year after birth for perinatal complications and infants’ health and nutrition status (ie, through home visit services).

Our study thus examines the effectiveness of a telehealth care intervention for improving women’s and infants’ care uptake in rural areas of Bangladesh. Additionally, it examines the effectiveness of telehealth care in detecting health problems among women and infants.

## Methods

### Study Design and Sites

**Figure 1:**
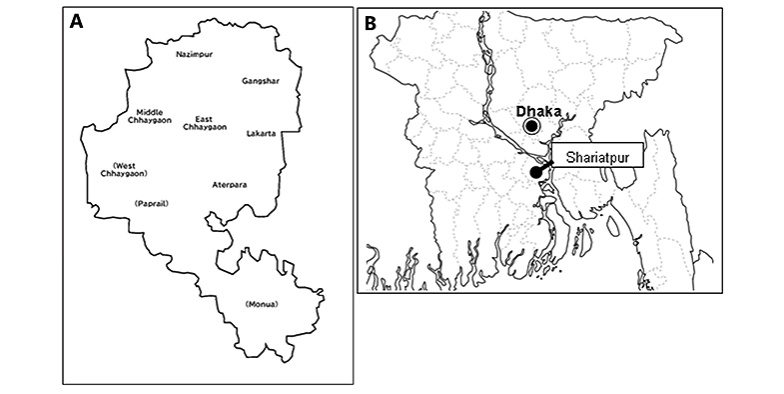
(A) Project site villages within the Chhaygaon Union of Shariatpur District. Among the nine villages, the six that are not bracketed are the study sites. (B) The location of Shariatpur District in Bangladesh.

Our study is quasi-experimental, and it will be conducted in 6 villages within the Chhaygaon Union of Bhedarganj Upazila (subdistrict), Shariatpur District, Bangladesh (Figure 1; trial registration number: ISRCTN44966621). Located approximately 80 km from Dhaka, these villages are in the suburb of the capital. Among the nine villages in the Chhaygaon Union, the East Chhaygaon, Middle Chhaygaon, and Lakarta villages were purposively set as the intervention cluster, and the Gangshar, Nazimpur, and Aterpara villages were set as the control cluster. The villages were allocated to the intervention and control areas such that the population sizes (intervention area: N=6378; control area: N=6178), geographic locations of the villages, and access to health facilities (each area has a health facility) were similar between the two areas. In the intervention arm, we conducted a pilot study in which we used the PHC system between June 2019 and May 2020 to follow women and infants from 16 gestational weeks to only 6 weeks post partum, with a shorter postpartum follow-up period than the one described herein.

### Study Population and Selection Criteria

**Figure 2:**
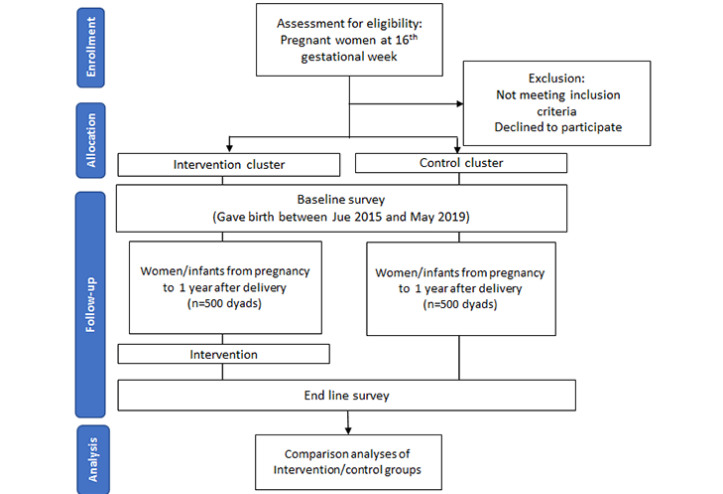
Flow diagram of participants for the enrollment, allocation, follow-up, and analysis stages.

A flow diagram of participants for the enrollment, allocation, follow-up, and analysis stages is presented in Figure 2. Different selection criteria will be applied to intervention, baseline survey, and end line survey participants. The inclusion criteria for intervention participants are (1) women at reproductive age (between 15 and 49 years), (2) women at ≥16 gestational weeks, and (3) women living in intervention villages at the time of enrollment. After birth, their infants will also be enrolled in the intervention cluster.

Baseline survey participants will be women in the intervention and control clusters who experienced giving birth between June 2015 and May 2019 (ie, before the pilot study). End line survey participants will be women in the intervention and control clusters who give birth between November 2020 and October 2024. Women who experience a miscarriage or a stillbirth during this period will also be included as participants.

### Recruitment and Consent of Participants

All eligible women living in the intervention villages will be recruited for the study intervention. Local informants will collect information about eligible women from local citizens every month in all of the intervention cluster villages by visiting resident reproductive-age women individually. For women who might be pregnant, a test kit will be provided to confirm their pregnancy status. The expected recruitment period for intervention participants is June 2020 to June 2024. Baseline survey participants will be recruited based on information regarding their past pregnancy experiences, which will be obtained through the local informants and individual home visits.

We will explain the study purpose to potential participants, and their consent will be indicated by their participation in the intervention study or the questionnaire surveys. Their participation will be voluntary, and they will be free to join or withdraw at any time.

### Interventions

We will conduct a telehealth care intervention for women (from pregnancy [at ≥16 gestational weeks] to 1 year after delivery) and their infants (from birth to 1 year of age). The content and processes of the interventions are detailed below.

#### The PHC-MNCH

We developed the PHC-MNCH system, which comprises a set of sensor devices that were selected based on international information standards and approved by Japanese pharmaceutical law. This sensor set also includes an Android tablet, internet router, and portable printer. The sensors include weighing scales, a digital sphygmomanometer, a blood glucose test sensor, a digital thermometer, pulse oximeters for adults and infants, a height measurement tape, a height measurement mat for infants, a blood hemoglobin meter, and urine test strips. The list of devices is presented in Multimedia Appendix 1.

#### Implementation Team

We created, in advance, a health care worker team that will perform health checkups on participants. The team consists of 2 local residents; one is a qualified paramedic, and the other is a traditional birth attendant. Using the PHC-MNCH system, the team members will act as mediators of health checkup implementation between participant women and physicians.

#### Telehealth Care Intervention

The health care worker team will visit women’s and infants’ homes to examine their health status by using the PHC-MNCH system and conducting interviews to check if they are experiencing any complications. All of the data will be inserted into the triage app installed on the tablet, which will automatically categorize the participants’ health statuses into the following four severity and color levels: healthy (green), cautious (yellow), affected (orange), and emergency (red). Finally, the health care worker team will print out the health checkup reports for women and infants. The women will be remotely connected to a medical physician stationed at Dhaka by using a video system, and they will receive the physician’s consultation on the basis of the results of their checkups, which will be sent through the app. If necessary, the physician will send prescriptions to the women via the health care worker team, using the same app.

#### Health Education

In addition to health checkups, the health care worker team will provide health education to mothers during each home visit through educational videos and brochures, with themes related to antenatal and postnatal care, danger signs in each pregnancy stage, and mothers’ and infants’ nutrition. The education materials were developed based on the regional context of Bangladesh, following a review by a local health expert. The education content is presented in Multimedia Appendix 2.

#### Maternal and Child Health Handbook

The collected health checkup data will be recorded in a physical maternal and child health handbook, so that the information can be kept for future reference. In addition to the health checkup records, this handbook contains health education information regarding perinatal danger signs, infants’ immunization records, and infants’ growth records. The handbook was developed based on the maternal and child health handbook that is presently used in Japan.

#### Health Checkups in the Control Area

Although we will not provide the PHC-MNCH telehealth checkup to the participants in the control area, they can receive any health checkups at any health facilities if they want, as general pregnant women, mothers, and children do in Bangladesh.

### Data Collection Methods and Evaluation

We will assess the impact of the intervention by comparing the clusters, using baseline and end line questionnaire survey data.

#### Baseline and End Line Survey

We will conduct the questionnaire survey at the baseline and end line periods for all eligible women in the intervention cluster and control cluster. The questionnaire items assess sociodemographic and economic status, the health services received and the times they were received, symptoms during pregnancy, and postpartum or neonatal complications. The PHC-MNCH researchers developed the survey in English, and the research assistants translated it into Bengali. A Bengali- and Japanese-speaking researcher then checked the accuracy of the translation.

#### Health Checkup

We will collect the health checkup data of intervention cluster participants throughout the intervention to identify gestational and maternal complication symptoms and health problems. The health checkups will be conducted during 9 home visits, which will be scheduled at approximately 16, 24, 32, and 36 weeks of gestational age and at approximately 2 to 3 days, 7 days, 6 weeks, 6 months, and 12 months after delivery or birth. The measurement- and interview-based checkup items are presented in Textboxes 1 and 2. Health statuses will be categorized into 4 levels according to the criteria developed by Japanese and Bengali obstetricians and medical professionals. Excerpts of criteria details are presented in Multimedia Appendix 3.

With regard to the control cluster, we will not collect health checkup biometric data. This is because conducting health checkups in the control area may affect the mothers’ awareness of health-seeking behaviors and result in bias in the intervention results.

**Health checkup items for mothers at different periods.**

**Measurements**

- All periods from the antenatal period to the motherhood period
  Weight, pulse, blood pressure, temperature, hemoglobin, urine protein, urine sugar, oxygen saturation, and blood glucose
- Additional measures for the antenatal period
  Fetal heartbeat, uterus height, edema, and baby’s position

**Interviews**

- Antenatal period
  Fetal movements, regular contractions, ruptures, vaginal bleeding, smelly vaginal discharge, headaches, vomiting, fevers, convulsions, and depressive symptoms
- Postnatal period
  Vaginal discharge, bleeding, uterus hardness, nutrition, nipple problems, perineum tear problems, wound infection, urinating problems, calf pain, headaches, shortness of breath, fevers, convulsions, and depressive symptoms
- Motherhood period
  Vaginal discharge, hematemesis, nutrition, nipple problems, headaches, shortness of breath, fevers, convulsions, depressive symptoms, urination problems, urinary leakages, and fecal leakages

**Health checkup items for infants at different periods.**

**Measurements**

- Both the after-birth period and the childhood period
  Weight, height, temperature, heart rate, oxygen saturation, cyanosis, and hemoglobin

**Mothers' interviews**

- After-birth period
  Breathing difficulties, feeding frequency, jaundice, pus, irritated cords, diarrhea, bleeding, and convulsions
- Childhood period
  Breathing difficulties, pus, diarrhea, hematemesis, convulsions, and the start of complementary feeding

### Study Outcomes and Measurements

The following outcomes will be compared based on the baseline and end line surveys: the frequency of antenatal and postnatal care visits by health care providers conducted through health checkups with the PHC or at health facilities; the detection rate of gestational complication symptoms (suspected gestational diabetes, pregnancy-induced hypertension, anemia, preterm birth, and postterm birth); the detection rate for neonates and infants with suspected jaundice, anemia, and growth delay; the percentage of infants who started complementary feeding at 6 months after birth; and the percentage of participants who sought health care or exhibited health-seeking behaviors or self-care behaviors upon experiencing health problems.

### Data Monitoring

Data managers will review the accuracy and completion of collected data and thereafter pool them in a project server. Verification checks will be performed to correct any discrepancies in records. Intervention implementation will be monitored by the field monitoring and research team members. All adverse events and unintended effects of the intervention will be reported monthly by the field monitoring team members and followed up by the research team members. Access to all monitoring-related information will be limited to the field monitoring and research team members.

### Participants’ Timelines

The details of participants’ timelines are described in Multimedia Appendix 4. Women will be enrolled at approximately 16 gestational weeks, that is, when they receive the first antenatal checkup. They will be followed up at 9 points in time via health checkups. Infants will be enrolled at birth and followed up at 5 points in time after birth.

### Sample Size

All eligible women and infants at the study sites will be included in our study. Approximately 1000 mother-infant dyads have been deemed eligible for this study among all clusters (500 dyads in each cluster). However, the minimum sample size for recruitment was calculated as 925 dyads for all clusters; 771 dyads in a cluster are required for analyses based on our pilot study, in which the percentage of antenatal care increased by ≥4 times (from 29% to 42%). A 2-tailed test will be conducted (power=0.95; α error=.05). A loss to follow-up rate of 20% was estimated.

### Statistical Analyses

First, we will conduct descriptive analyses of and comparison tests between the intervention and control clusters to assess their similarities. Second, to evaluate the effectiveness of the intervention and compare the clusters, we will perform a multilevel logistic regression model of end line surveys, adjusting for the effects of clustering. Finally, sensitivity analyses will be conducted to assess the robustness of the model. The statistical significance will be set at a *P* value of <.05. All data analyses will be performed by using IBM SPSS (IBM Corporation).

### Ethics Approval

Our study was approved by the institutional review boards of Kyushu University (approval number: 20202021). Written informed consent will be obtained from all participants. Participation will be voluntary, and confidentiality will be maintained. Participants can withdraw from the study for any reason at any time. The intervention will be introduced to the control cluster after the study is completed.

All of the information obtained in our study will remain confidential. Access to information will be limited to the health care workers and data entry management staff for the duration of the study. Research records will be identified only by study ID numbers.

### Dissemination

The results of our study will be disseminated through peer-reviewed journals and international conferences. Additionally, the telehealth care system will be disseminated to other areas in Bangladesh through the collaboration of local communities and research collaborator organizations. Important protocol changes will be communicated to the research ethics committee and the clinical trials registry.

Designing or reporting will not involve patients of the public. However, residents will be involved in interventions, serving as health care workers or local informants. The dissemination plan will be discussed with the local community and research collaborator organizations.

## Results

Recruitment began in June 2020, and it is expected to continue until June 2024. We consented 295 mothers in the study by June 2022. Data cleaning and analysis will begin after the data collection is complete.

## Discussion

In our study, the intervention arm participants are expected to have better health statuses than those of the control arm, as they will receive care through the PHC-MNCH. Our community-based intervention study is extremely important for realizing the continuum of care in MNCH through a telehealth care system and evaluating the improvement of mothers’, newborns’, and children’s health status. Previous studies that investigated the effectiveness of the continuum of care in MNCH mostly focused on the perinatal period [20,21]. Through our trial, we will attempt to show whether the continuum of care, which extends from the antenatal period to the motherhood and childhood periods, is effective in improving the health of mothers and infants. The study will further accumulate evidence on the effectiveness of the continuum of care.

Our proposed study also has a limitation. The intervention sites include some of the areas where we conducted the pilot study. Therefore, some residents of the intervention sites may have a better understanding of the continuum of care than those in the control group. However, since the participants in the pilot study are not included in the proposed study, the effectiveness of the intervention will be evaluated based on its status prior to the pilot study. As such, we believe that this limitation can be controlled.

## Appendix

Multimedia Appendix 1 Device specifications.

Multimedia Appendix 2 Health education content.

Multimedia Appendix 3 Excerpts of criteria for antenatal checkup.

Multimedia Appendix 4 Participant timeline of the Portable Health Clinic for Maternal, Newborn, and Child Health intervention study.

## Abbreviations

MNCH: maternal, newborn, and child health
PHC: Portable Health Clinic
PHC-MNCH: Portable Health Clinic for Maternal, Newborn, and Child Health

## Data Availability

The data that have been generated and analyzed during our study are available from the corresponding author on reasonable request.
