# Supplementary material for: Telehealth Care for Mothers and Infants to Improve the Continuum of Care: Protocol for a Quasi-Experimental Study
Source: JMIR Res Protoc. 2022 Dec 15;11(12):e41586. doi: 10.2196/41586 (PMC9801263; doi:10.2196/41586)
Supplement: Multimedia Appendix 1 [file resprot_v11i12e41586_app1.docx]

**Appendix 1. Portable Health Clinic for Maternal and Child Health**

|  | **Devices Set Specifications** |  |
| --- | --- | --- |
| Device and sensor | Manufacturer | Country |
| Weighing scale | Omron Corporation | Japan |
| Sphygmomanometer | A&D Company Ltd | Japan |
| Blood glucose test sensor | Terumo Corporation | Japan |
| Digital thermometer | Omron Corporation | Japan |
| Pulse oximeter (adult) | Matsuyoshi & Co., Ltd | Japan |
| Pulse oximeter (infant) | Matsuyoshi & Co., Ltd | Japan |
| Height measurement tape | Seca GmbH & Co. KG | Germany |
| Height measurement mat | Seca GmbH & Co. KG | Germany |
| Haemoglobin meter | Hemocue | Sweden |
| Urine test strip | Terumo Corporation | Japan |
