# Supplementary material for: Telehealth Care for Mothers and Infants to Improve the Continuum of Care: Protocol for a Quasi-Experimental Study
Source: JMIR Res Protoc. 2022 Dec 15;11(12):e41586. doi: 10.2196/41586 (PMC9801263; doi:10.2196/41586)
Supplement: Multimedia Appendix 2 [file resprot_v11i12e41586_app2.docx]

**Appendix 2. Health Education Content**

**Education Timings Education Content 16 gestational weeks** • Pregnancy and care

- Importance of antenatal check-up
- Self-care during pregnancy
- Danger signs in pregnancy
- Preparedness during pregnancy

**24 gestational weeks** • Importance of antenatal check-up

- Self-care during pregnancy
- Danger signs in pregnancy
- Preparedness during pregnancy

**32 gestational weeks** • Importance of antenatal check-up

- Self-care during pregnancy
- Danger signs in pregnancy
- Preparedness during pregnancy
- Choices of delivery place

**36 gestational weeks** • Self-care during pregnancy

- Danger signs in pregnancy
- Preparedness during pregnancy
- Choices of delivery place
- Preparations for delivery if occurs at home
- Danger signs during delivery
- Essential newborn care

**Delivery/birth** • Essential newborn care

- Danger signs in newborns
- Postnatal care
- Newborn care

**2-3 days** • Danger signs in newborns

- Postnatal care
- Newborn care
- Danger signs during postnatal period
- Self-care during postnatal period
- Care of newborns
- Nutrition

**7 days** • Danger signs in newborns

- Postnatal care
- Newborn care
- Danger signs during postnatal period
- Self-care during postnatal period
- Care of newborns
- Nutrition

**6 weeks** • Danger signs during postnatal period

- Self-care during postnatal period
- Care of newborns
- Nutrition

**6 months** • Nutrition

**12 months** • Nutrition
