# Supplementary material for: Telehealth Care for Mothers and Infants to Improve the Continuum of Care: Protocol for a Quasi-Experimental Study
Source: JMIR Res Protoc. 2022 Dec 15;11(12):e41586. doi: 10.2196/41586 (PMC9801263; doi:10.2196/41586)
Supplement: Multimedia Appendix 3 [file resprot_v11i12e41586_app3.docx]

2022.6.1

Excerpts of criteria for antenatal check-up

| **Stage** | **Check-up Item** | | **Unit** | **Lower Warning** | **Healthy (green)** | **Caution (yellow)** | **Affected (orange)** | **Emergent (red)** | **Upper Warning** |
| --- | --- | --- | --- | --- | --- | --- | --- | --- | --- |
|  | Expected delivery date | |  |  | Calculation: 280 days to the first day of last menstrual period | | | |  |
|  | Blood Pressure | |  | <70 | <130 |  | ≧130 & ≦139 | ≧140 | >220 |
|  |  |  |  | <50 | <80 |  | ≧80 & ≦89 | ≧90 | >140 |
|  | Pulse (BP) | | bpm | <50 | >=60 & <100 | >= 50 & <60 or  >=100 & <120 | <50 OR >=120 |  | >130 |
|  | Temperature (dec) | | ℃ | <33.0 | <37.0 | >=37.0 & <37.5 | >=37.5 & <38.0 | ≧38.0 | >40.0 |
|  | Hemoglobin (dec) | | g/dl | >18.0 | ≧11 | <11 & ≧10 | <10 & ≧8 | <8.0 | <6.0 |
|  | Urine Protein | |  |  | Negative | ± | ＋ | ++ or +++ |  |
|  | Urine Sugar | |  |  | Negative | ± | ＋ | ++ or +++ |  |
|  | Ask mother her condition | Movement of baby |  |  | Yes |  | Yes |  |  |
|  |  | Regular contractions |  |  | No |  | Yes |  |  |
|  |  | Rupture |  |  | No |  | Yes |  |  |
|  |  | Vaginal bleeding |  |  | No |  | Yes |  |  |
|  |  | Smelly vaginal discharge |  |  | No |  | Yes |  |  |
|  |  | Severe headache with blurred vision |  |  | No |  | Yes |  |  |
