# Supplementary figures and images for: Telehealth Care for Mothers and Infants to Improve the Continuum of Care: Protocol for a Quasi-Experimental Study
Source: JMIR Res Protoc. 2022 Dec 15;11(12):e41586. doi: 10.2196/41586 (PMC9801263; doi:10.2196/41586)

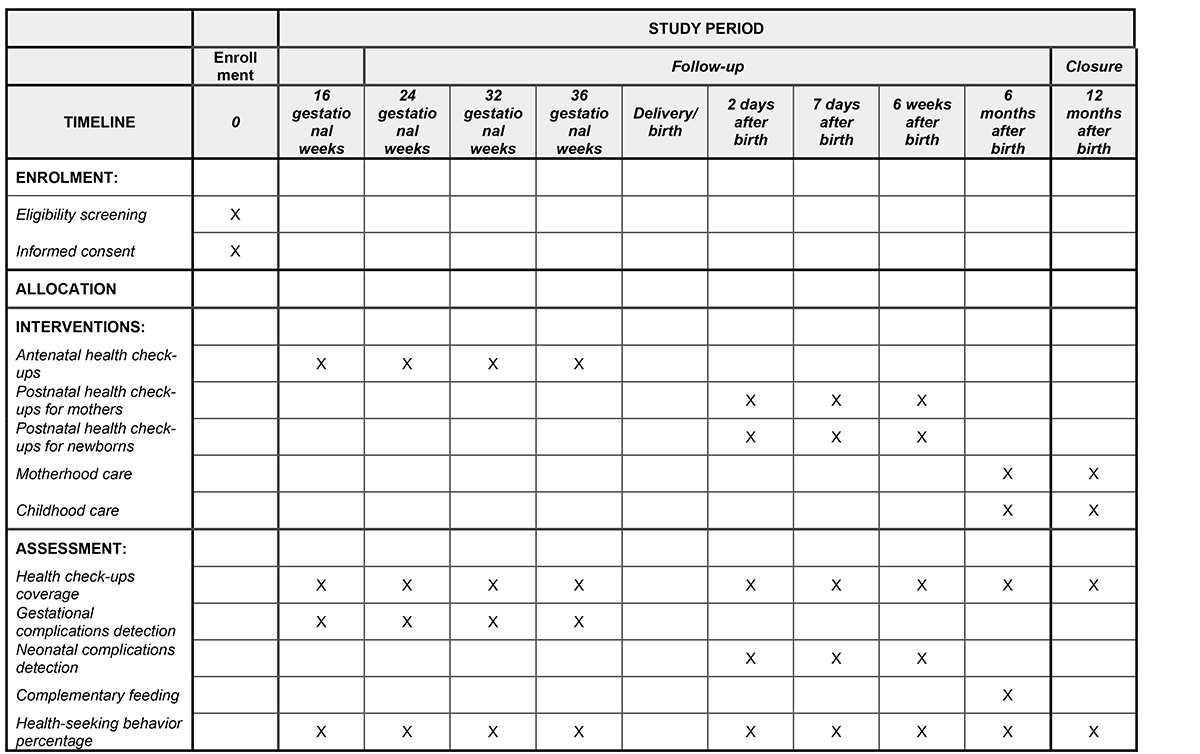

Supplement: Multimedia Appendix 4 [file resprot_v11i12e41586_app4.png]
